# AI-assisted digital tooth arrangement system for esthetic optimization in virtual dentures

**DOI:** 10.6026/973206300220846

**Published:** 2026-02-28

**Authors:** Karthikeyan Vasudevan, Vijay Parmar, Gouri R Reddy, Ankit Dhimole, Ekta Chheda, Deepali Neeraj

**Affiliations:** 1 Department of Prosthodontics, SRM Dental College, Ramapuram, Chennai, India; 2 Department of Dentistry, AIIMS Rajkot, Gujarat, India; 3 Department of Dentistry, Shri Atal Bihari Vajpayee Medical College and Research Institute, Bengaluru, India; 4 Department of Oral Medicine & Radiology, Hitkarini Dental College & Hospital, Jabalpur, Madhya Pradesh, India; 5 Department of Prosthodontics, Crown and Bridge, Government Dental College and Hospital, Ahmedabad, India; 6 Department of Prosthodontics and Crown and Bridge, Sharda University, School of Dental Sciences, Uttar Pradesh, India

**Keywords:** Artificial intelligence, full denture, digital dentistry, tooth arrangement, dental esthetics

## Abstract

Artificial tooth arrangement in complete dentures is a subjective and time-consuming process largely dependent on operator experience. An artificial intelligence-aided digital tooth arrangement system using a convolutional neural network trained on 2,500 clinically validated denture cases was developed and evaluated. Forty edentulous patient datasets were arranged using both AI-assisted and conventional digital workflows and assessed by prosthodontists and lay evaluators. The AI system significantly reduced arrangement time and achieved equal or superior esthetic scores in parameters such as midline accuracy and proportional harmony, while lay attractiveness ratings were comparable. Thus, we show that AI-assisted tooth arrangement offers an efficient, reliable and esthetically predictable approach for standardizing digital complete denture design.

## Background:

Total edentulism still has a high percentage of the world population especially among the elderly where it has serious consequences to masticatory, phonetics, face esthetics and psychological wellbeing [1]. Although there is improvement in the implant dentistry, conventional complete dentures still continue to be the major mode of treatment of most of the edentulous patients because of medical, anatomical, or financial reasons [2]. To achieve successful rehabilitation of edentulous patients, functional restoration is not enough, but special attention must be paid to esthetic results that meet the expectations of individuals and help to improve the quality of life [3]. One of the most significant and technical stages of the fabrication of dentures is the position of the artificial teeth in full-dentures. This is whereby each tooth is placed in line with the concepts of the occlusion, phonetics and esthetics and the individual anatomical features of the patient [4]. Conservative methods of placing teeth have greatly depended on the clinical sense and artistic instinct of dental technicians and this has led to a lot of variation in the results based on the experience of the operator and subjective interpretation of esthetic rule [5]. The rules of esthetic composition of anterior teeth include many related considerations such as tooth size, shape and color choice, mid-line placement, smile line arrangement, incisal plane direction and creation of suitable vertical and horizontal overlaps [6]. Other factors are the reproduction of the natural properties of the teeth like subtle rotations, tilting and differences in spacing that create an artificial homogenous look [7]. The combination of these several parameters when preserving the functional harmony is a complicated optimization problem which, in the past, relied on the intuition and experience of experienced professionals. The computer-aided design and manufacturing technologies that the digital revolution in the field of prosthodontics has brought about have a greater level of precision, reproducibility and efficiency than the traditional methods in the laboratory procedures [8]. Digital denture workflows allow the virtualization of artificial teeth in specific software settings, it is possible to visualize, modify and optimize them in real-time and then print them and then bend them physically [9]. Nevertheless, even modern digital denture systems still cannot afford much automation in placement of individual teeth and as such they continue to necessitate subjective decision-making by operators, the hallmark of the conventional methods [10].

The artificial intelligence technologies have shown a very impressive performance in pattern recognition, prediction as well as optimization in various fields such as in medical and dental applications due to the deep learning algorithms that are built upon the neural network architecture [11]. In the field of dentistry, AI systems have been effectively used in diagnostic imaging interpretation, treatment planning and prediction outcome with precision few to none lower than that of human specialists with regard to particular tasks [12]. The utilization of these technologies in the processes of designing prosthetic treatment is a natural development that would help overcome the subjectivity and fluctuation of the existing approaches to tooth ordering [13]. Recent research has examined the use of AI in different fields of dental prosthetics, such as automated tooth segmentation, shade matching and optimization of the crown design [14]. The initial research on AI-aided denture design has already shown the possibility to automate some of the workflow, but there has been little comprehensive research to cover all the teeth in the arrangement to maximize esthetics despite the promise [15]. It is possible that the tacit knowledge of the experienced practitioners might be captured and standardized by developing AI algorithms that might be trained on large datasets of clinically proven arrangements [16]. The esthetic assessment of dental prostheses is even more complicated by the fact that the sense of beauty is subjective in nature and people differ in their tastes. Though professional tests in accordance with well-known esthetic principles offer more organized evaluation systems, the patient and layperson views might not be consistent with those of experts [17]. The overall critique of AI-generated arrangements must thus be conducted by considering a wide range of perspectives such as professional and nonprofessional ones [18]. Although the use of AI tools in dental prosthetics is gaining attention, the literature is significantly lacking in the research and clinical confirmation of complete AI-assisted tooth arrangement frameworks. The current literature has concentrated more on individual elements of the design process instead of wholesome systems that can produce entire esthetic design arrangements [19]. In addition, stringent testing of AI-generated designs versus traditional designs created by an expert based on some standardized assessment criteria is not provided [20]. Therefore, it is of interest to create and test an AI-supported computerized tooth arrangement system to esthetically optimize virtual complete dentures. These objectives of the study were: (1) convolutional neural network trained on verified clinical data to predict optimal tooth position, (2) comparing AI-trained arrangements to traditional digital arrangements made by skilled dental technicians and (3) comparing the resultant arrangements against professional esthetic criteria and lay person attractiveness ratings. The hypothesis of the research was as follows: AI-assisted arrangement would demonstrate similar esthetic results to traditional digital arrangement and save design time substantially.

## Materials and Methods:

## Study design:

The proposed comparative prospective study took place in the Digital Prosthodontics Research Center between January 2023 and October 2024. The study was approved by institutional ethics committee ( Protocol Reference: DPRC-2023-0124) and carried out on the principles of the Declaration of Helsinki. Every participant gave an informed written consent beforehand.

## AI system development:

The tooth arrangement system with the help of AI was created with the convolutional neural network architecture with ResNet-50 with specific adjustments when used in dentistry. The training data was a sample of 2,500 complete denture cases of five academic dental centers with the relevant data sharing arrangements. Cases were chosen by documented clinical success which is demonstrated by patient satisfaction ratings of over 80 percent on validated questionnaires and a rating by the prosthodontist of acceptable or optimum esthetic results.

## The neural network input parameters were:

[1] STL scans of maxillary, mandibular arches (three-dimensional).

[2] Facial reference photographs (frontal, lateral and smile)

[3] Cephalometric landmarks and measurements.

[4] Inter-arch relationship recording.

[5] Demographic information about the patient (age, gender, the type of facial form)

The network was trained to provide the best three-dimensional coordinate and angulation of each of the 28 artificial teeth according to the concept of esthetic principles which include golden proportion relationships, tooth-to-face width ratios, midline placement with respect to the facial landmarks and smile arc arrangement. The data was augmented to improve the generalization of models. The training was done under TensorFlow 2.10 framework on NVIDIA A100 GPUs clusters between the following hyperparameters: |human|>The training was conducted using the following parameters: learning rate 0.0001, batch size 32, Adam optimizer and 200 training epochs. The data was split into training (70 percent), validation (15 percent) and testing (15 percent) data. Optimization of model performance was conducted in terms of mean positional deviation against proved clinical settings on the test data.

## Patient sample selection:

The prospective evaluation of the AI system was done by recruiting forty edentulous patients who were going to have complete dentures made at the university prosthodontics clinic.

## Inclusion criteria:

[1] Maximally and or mandibular arches full edentulism.

[2] Age 50-80 years

[3] Sufficient ridge residual anatomy to support standard denture.

[4] None of full denture experience or dissatisfaction with current dentures.

[5] Eagerness to be involved in esthetic evaluation processes.

## Exclusion criteria:

Severe ridges requiring preprosthetic surgery.

[1] Major maxillomandibular skeletal imbalance.

[2] Oral mucosal active pathology.

[3] Impaired informed consent Cognitive impairment.

[4] Pre-trauma or surgery to facial symmetry.

## Digital data acquisition:

All enrolled patients were subjected to standardized acquisition of digital data. By means of a calibrated intraoral scanner (TRIOS 4, 3Shape, Copenhagen, Denmark) a calibration in accuracy specifications, intraoral impressions were taken. The scans of the faces were taken on a structured light facial scanner (Face Hunter, Zirkonzahn, Gais, Italy) with the patients placed in natural head position as well as relaxed position of the lips. The conventional techniques were used to get the jaw relation records which were subsequently digitized through the bite registration feature on the scanner. The measurements were in the form of Cephalometric analysis based on the lateral cephalograms, either based on previous diagnostic records or taken specifically to the study protocol. All computer-generated data were fed into the self-written AI set-up software platform and the commercial digital denture model software (DentalCAD, exocad, Darmstadt, Germany) to conventional arrangements.

## Arrangement of teeth protocols:

This means that the mentor will support the arrangement in an AI-assisted manner (Experimental Group). The trained neural network was used to process digital datasets that produced the initial positions of teeth in all 28 artificial teeth. Each tooth position had system output which was in 3-dimensional coordinates (x, y, z) and the angulation (mesio-distal, labio-lingual, rotational). It was automatically generated in the custom software environment and no interaction with operators allowed.

## Traditional digital arrangement (Control Group):

Similar digital data were given to three qualified dentist technicians (over 10 years of full denture experience) who individually ran tooth arrangements with commercial denture programs. Technicians were not allowed to see AI-generated arrangements and were guided by existent esthetic guidelines according to the standardized protocol document. The patient data sets were randomly allocated to each technician. The protocols time of arrangement was recorded as the time that it took to have the tooth positioned.

## Esthetic evaluation:

## Professional evaluation:

Professional evaluators were ten board-certified prosthodontists who had at least 5 years of clinical practice. Before assessment, all the evaluators were trained on calibration using reference images and scoring criteria. The evaluators were not informed of the method of arrangement, but blindly rated virtual denture arrangements presented on calibrated monitors with a validated esthetic scoring instrument.

The assessment tool included six parameters which are measured in 5-point Likert scales:

[1] Central alignment (central to facial midline)

[2] Harmony of the smile line (relative to the curvature of the lips)

[3] Tooth ratios (suitable ratios of size)

[4] Proportional harmony (golden proportion approximation)

[5] Natural appearance (absence of superficial homogeneity)

[6] General overall quality of esthetic rating (global rating)

As well, rating of each arrangement was done by rating it by its clinical acceptability (acceptable/requires modification/ unacceptable).

## Lay person evaluation:

Smile attractiveness was measured on thirty lay evaluators (15 males, 15 females, age 25-65 years) with no dental history. Each set of rendered smile images was rated by the evaluators on overall attractiveness on a visual analog scale (0-100 mm, anchored between very unattractive and very attractive).

## Statistical analysis:

The statistical analyses were calculated with the help of IBM SPSS Statistics version 28.0 (IBM Corporation, Armonk, NY, USA) and the R version 4.2.2 (R Foundation, Vienna, Austria). The calculation of sample size was performed to identify a difference of the mean of 0.5 points on the 5-point esthetic scale with a standard deviation of 0.7, alpha = 0.05 and power = 0.85 which gave the required minimum sample size as 34 cases. Shapiro-Wilk tests were used to test the normality of the continuous variables. Within-subject comparisons of the arrangement methods were performed by paired t-tests or the use of Wilcoxon signed-rank tests. Between-group comparisons were done using independent samples t-tests where it was applicable. Inter-rater reliability was determined by use of intraclass correlation coefficients (ICC) two way random effects model. The evaluation of agreement on clinical acceptability was performed at Fleiss coefficient of kappa. The Pearson correlation coefficients were to be used to compare relationships among professional and lay evaluator ratings. The descriptive statistics were reported by means of standard deviation in the form of mean and standard deviation in the form of frequencies in percentages as frequency distributions of the variables that were continuous and nominal respectively. All analyses were set to be statistically significant at p<0.05.

## Results:

All forty enrolled patients completed study procedures without dropout. The sample comprised 23 females (57.5%) and 17 males (42.5%) with mean age of 64.8±8.3 years (range: 51-78 years). Arch configurations included 18 maxillary complete dentures (45%), 6 mandibular complete dentures (15%) and 16 bilateral complete dentures (40%). The AI system successfully generated tooth arrangements for all datasets within the established parameters. Mean processing time for AI-assisted arrangement was 18.4±4.2 minutes, representing a 61.1% reduction compared to conventional digital arrangement time of 47.3±11.6 minutes (p<0.001). The processing time difference remained significant across all arch configurations (Table 1). Inter-rater reliability among professional evaluators was excellent for both arrangement methods (ICC=0.87 for AI-assisted, ICC=0.84 for conventional), indicating consistent assessment across evaluators. Professional evaluators rated AI-assisted arrangements significantly higher than conventional arrangements for midline accuracy (4.52±0.48 versus 4.18±0.62, p=0.008) and proportional harmony (4.38±0.54 versus 3.96±0.71, p=0.004) (Table 2). No significant differences were observed for smile line harmony, tooth proportions, natural appearance, or overall esthetic quality, though AI-assisted arrangements showed numerically higher scores across all parameters. Analysis of clinical acceptability ratings demonstrated that 95.0% of AI-assisted arrangements were rated as acceptable without modification compared to 87.5% of conventional arrangements.

The proportion of arrangements requiring modification was 5.0% for AI-assisted versus 10.0% for conventional, with 2.5% of conventional arrangements rated as unacceptable compared to 0% for AI-assisted arrangements. These differences approached but did not reach statistical significance (χ^2^=3.42, p=0.064). Subgroup analysis by arch configuration revealed that AI-assisted arrangements demonstrated greatest advantage in bilateral complete denture cases, where coordination of maxillary and mandibular tooth positions is most complex. For bilateral cases, overall esthetic quality scores were 4.42±0.51 for AI-assisted versus 3.98±0.68 for conventional arrangements (p=0.024). Lay evaluators demonstrated high inter-rater reliability (ICC=0.81) in attractiveness assessments. Mean attractiveness ratings on the visual analog scale showed no significant difference between AI-assisted (68.4±12.3 mm) and conventional (66.8±14.1 mm) arrangements (p=0.412) (Table 3). nAnalysis by evaluator demographics revealed no significant influence of evaluator age or gender on attractiveness ratings for either arrangement method. However, younger evaluators (age <45 years) demonstrated slightly higher correlation between their ratings and professional overall esthetic quality scores (r=0.68) compared to older evaluators (r=0.54). The correlation between professional overall esthetic quality ratings and lay person attractiveness ratings was moderate and significant for both AI-assisted (r=0.62, p<0.001) and conventional (r=0.58, p<0.001) arrangements, suggesting reasonable alignment between professional and lay perspectives on esthetic quality. Subgroup analysis by smile characteristics revealed that arrangements featuring wider smile arcs and greater tooth display received higher attractiveness ratings from lay evaluators regardless of arrangement method, confirming the importance of these factors in public perception of denture esthetics.

## Discussion:

As it can be seen in the current study, an AI-supported tooth arrangement platform can generate esthetically pleasing virtual denture designs and with a much shorter processing duration than traditional digital processes. The base of the 61 percent decrease in the arrangement time and similar or even better esthetic results, implies that there is a great deal of workflow optimization potential in digital prosthodontic practice [21]. Largely, finding the superiority of the AI-assisted arrangement of midline accuracy is especially remarkable. Precise midline positioning is one of the major esthetic criteria that affect the overall perception of smiles largely, but it is difficult to bring the manual positioning near the face landmarks accurately [22]. The variability in this critical parameter seems to be increased by the capability of the AI system with the capability of integrating facial reference data and optimizing automatically the position of the midline basing on the previously tested cases. The higher proportional harmony scores in the arrangements by AI could be attributed to the ability of the system to apply the mathematical relationships like ratio of the golden proportions that are hard to apply exactly by using the manual method [23]. Although, intuitive knowledge of desirable proportions is gained through experience, the numerical aspect of AI-based optimization can offer greater compliance with laid down proportional standards through a wide spectrum of patient manifestations. The fact that the difference in the scores of the natural appearances of the methods is not significant deserves attention. To make natural-looking arrangements, the tooth positioning needs a slight variation to avoid an artificially even look on the dentures that is a result of bad design [24]. The AI system used randomized micro- perturbations to differentiate the dentitions according to the patterns noted in natural ones, albeit the scale and distribution of perturbations might need additional adjustment to attain more realistic results. The similarity in the ratings of attractiveness of the lay evaluators to the two arrangement modes indicates that AI-generated designs satisfy the standards of the lay population on the aesthetics of dentistry, even with the grossly shorter design time [25]. This result has a significant implication on clinical efficiency, in that patient-perceived outcomes are the final outcome of a successful prosthetic. The moderate consistency in the relationship between professional and lay ratings supports that the main concepts of technical esthetics tend to correspond with the common sense even though there is a certain variation in preferences to aesthetics among people. The clinical acceptability level of the AI-assisted arrangements (95 percent) shows that the system generates designs that can be used in a clinical application (without the need to necessarily change in the majority of cases) [26]. This reliability might be useful in workflow models, whereby AI-generated arrangements are a final design of simple cases and human supervision and modification are applied to complex cases that need customized adjustment.

The benefit of AI-aided arrangement with regard to time is of great practical value to the clinical workflow and lab productivity. The decrease of about 47 minutes down to 18 minutes per case is a huge saving in time that would lead to increased capacity of the services and may lead to a decrease in the cost of treatment [27]. Moreover, the fact that it takes the same amount of processing time irrespective of whether an operator is available also handles the workforce constraint, which can limit the productivity of the laboratory. The specified benefit of AI-assisted setups in bilateral complete denture cases deserves specific focus. One of the most challenging factors when it comes to complete denture design is the coordination of the two, maxillary and mandibular tooth positions, so that they combine to provide harmonious esthetic and functional results [28]. The fact that the AI system can optimize two arches at the same time on inter-arch relationships can have some benefits compared to the sequential arrangement of arches manually. The nature of the training data has a significant effect on the performance and generalization of the system of AI. The existing system was also conditioned on cases in academic institutions according to the conventional rules of esthetic, which can restrict it to other populations or cultural backgrounds with alternative esthetic orientations [29]. Another vital direction of system development is the expansion of training datasets to include more diversity in the facial morphology, arch anatomy and cultural esthetic preferences. There are a few methodological issues that deserve a mention when deriving out these conclusions. The test was done on virtual representations and not the fabricated denture that had been tested clinically but this might not be a full-scale esthetic outcomes at the actual clinical environment [30]. The study also evaluated the results of initial arrangement and made no further evaluation of the effectiveness of later adjustment of occlusiveness and feedback changes made by patients that usually improve final prosthetic designs. The professional assessors are experienced and calibrated; however, there could be personal preferences, which made them score differently although the criteria were not personalized. Although the sample of lay evaluator is demographically differentiated, it might not be representative of the general population preferences towards dental esthetics. Moreover, the represented single AI system architecture and training protocol can be inadequate in terms of performance capabilities of other algorithmic strategies. The clinical validation studies that will involve investigating patient satisfaction with the fabricated dentures designed with the help of AI but based on the traditional workflow should also be conducted in the future [31]. The clinical application could be improved by the research that investigates the possibilities of AI systems customization to reflect personal preferences and cultural esthetic diversity. Also, the hybrid workflows involving AI-generated starting layouts and human optimization can be investigated to maximize the balance between efficiency and individualization [32].

## Conclusion:

We show that an AI-assisted digital tooth arrangement system is a reliable and efficient approach for esthetic optimization in virtual complete dentures, achieving a 61% reduction in arrangement time while producing esthetically acceptable designs in 95% of cases. AI-assisted arrangements showed superior midline accuracy and proportional harmony compared to technician-driven workflows, indicating improved consistency in the application of key esthetic principles. Overall esthetic ratings by professionals and laypersons were comparable between methods, supporting the clinical potential of AI-aided denture design while highlighting the need for further validation through patient-centered and clinical outcome studies.

## Figures and Tables

**Table 1:** Arrangement time and processing characteristics by method

**Parameter**	**AI-Assisted**	**Conventional**	**Difference**	**p-value**
	**Mean ± SD**	**Mean ± SD**	**(%)**	
Overall Processing Time (min)	18.4 ± 4.2	47.3 ± 11.6	-61.10%	<0.001*
**By Arch Configuration**				
Maxillary only (n=18)	16.2 ± 3.8	42.1 ± 9.4	-61.50%	<0.001*
Mandibular only (n=6)	14.8 ± 2.9	38.6 ± 7.8	-61.70%	<0.001*
Bilateral (n=16)	22.4 ± 4.1	56.8 ± 12.2	-60.60%	<0.001*
**Technical Metrics**				
Successful generation rate (%)	100	100	0	-
Cases requiring restart (n)	0	3	-	-
Mean iterations per case	1.0 ± 0.0	2.4 ± 1.1	-	<0.001*
*Paired t-test;
SD: Standard deviation

**Table 2:** Professional esthetic evaluation scores by arrangement method

**Evaluation Parameter**	**AI-Assisted**	**Conventional**	**Mean Difference**	**p-value**
	**Mean ± SD**	**Mean ± SD**	**(95% CI)**	
Midline Accuracy	4.52 ± 0.48	4.18 ± 0.62	0.34 (0.09-0.59)	0.008*
Smile Line Harmony	4.28 ± 0.56	4.12 ± 0.64	0.16 (-0.11-0.43)	0.241
Tooth Proportions	4.18 ± 0.62	4.02 ± 0.58	0.16 (-0.10-0.42)	0.218
Proportional Harmony	4.38 ± 0.54	3.96 ± 0.71	0.42 (0.14-0.70)	0.004*
Natural Appearance	4.08 ± 0.68	3.94 ± 0.72	0.14 (-0.17-0.45)	0.368
Overall Esthetic Quality	4.32 ± 0.52	4.08 ± 0.66	0.24 (-0.03-0.51)	0.078
**Clinical Acceptability n (%)**				
Acceptable	38 (95.0)	35 (87.5)	-	0.064†
Requires Modification	2 (5.0)	4 (10.0)	-	
Unacceptable	0 (0.0)	1 (2.5)	-	
*Paired t-test;
†Chi-square test;
SD: Standard deviation;
CI: Confidence interval Scale:
1=Poor, 2=Fair, 3=Acceptable,
4=Good, 5=Excellent

**Table 3:** Lay person attractiveness ratings by arrangement method

**Parameter**	**AI-Assisted**	**Conventional**	**Difference**	**p-value**
	**Mean ± SD**	**Mean ± SD**		
Overall VAS Score (mm)	68.4 ± 12.3	66.8 ± 14.1	1.6	0.412
**By Evaluator Demographics**				
Female evaluators (n=15)	69.2 ± 11.8	67.4 ± 13.6	1.8	0.387
Male evaluators (n=15)	67.6 ± 12.9	66.2 ± 14.7	1.4	0.524
Age <45 years (n=12)	70.8 ± 10.4	68.2 ± 12.8	2.6	0.318
Age≥45 years (n=18)	66.8 ± 13.4	65.8 ± 15.1	1	0.642
**Rating Distribution n (%)**				
High (≥75 mm)	18 (45.0)	15 (37.5)	-	0.264†
Moderate (50-74 mm)	19 (47.5)	20 (50.0)	-	
Low (<50 mm)	3 (7.5)	5 (12.5)	-	
**Correlation Analyses**				
Professional rating correlation (r)	0.62	0.58	-	-
p-value	<0.001	<0.001	-	-
*Paired t-test;
†Chi-square test;
VAS: Visual analog scale;
SD: Standard deviation
